# Crosslinked Zwitterionic PVA-*g*-SBMA/PEDOT:PSS Networks for Mechanically Robust All-Solid-State Electrolytes

**DOI:** 10.3390/polym18030343

**Published:** 2026-01-28

**Authors:** Chia-Wen Wei, Chia-Yu Chen, Shyh-Chyang Luo, Dmitry G. Belov, Szu-Nan Yang

**Affiliations:** 1 Department of Materials Science and Engineering, National Taiwan University, No. 1, Sec. 4, Roosevelt Road, Taipei 10617, Taiwan; tiffanywei0305@gmail.com (C.-W.W.); osukon8@gmail.com (C.-Y.C.); shyhchyang@ntu.edu.tw (S.-C.L.); 2 Prologium Innovation Europe, R&D Center, 6 Rue de la Terre de Fue, 91940 Les Ulis, France; 3 Prologium Technology Co., Ltd., No. 6-1, Ziqiang 7th Rd., Zhongli Dist., Taoyuan City 320, Taiwan

**Keywords:** solid polymer electrolytes, zwitterionic, PVA-*g*-SBMA, crosslinking, PEDOT:PSS

## Abstract

Conventional lithium-ion batteries face issues like electrolyte leakage and interface instability. Solid-state lithium batteries with solid electrolytes address these, while solid-state polymer electrolytes (SPEs) offer safety and flexibility. This study primarily aimed to develop and synthesize a graft copolymer, PVA-*g*-SBMA, which was successfully synthesized by grafting [2-(methacryloyloxy)ethyl] dimethyl-(3-sulfopropyl)ammonium hydroxide (SBMA) onto poly(vinyl alcohol) (PVA). PVA provided excellent film-forming ability and mechanical strength, enhancing flexibility and stability in solid-state devices. Meanwhile, SBMA’s zwitterionic structure promoted efficient ion transport, improving ionic conductivity and solid electrolyte performance in energy storage applications. From the results, the proton assignment of the PVA-*g*-PSBMA zwitterionic graft copolymers was investigated via ^1^H NMR spectra. The molecular weight of the graft copolymer was determined through aqueous GPC; the number average molecular weight (Mn) was 15,755, and the PDI was 1.17. The grafting efficiency of SBMA was calculated as 25%. However, the material lacked sufficient mechanical properties, leading to brittle membranes. To address this issue, we crosslinked the film to improve its mechanical properties. The grafted copolymer was crosslinked with the PEDOT:PSS as a crosslinkable sulfonated component using (3-glycidyloxypropyl)trimethoxysilane (GOPS) as the crosslinker and dimethyl sulfoxide (DMSO) as solvent to complete the crosslinking reaction. The crosslinking mechanism involved the reaction between hydroxyl groups on PVA and PSS, while the GOPS bonded with PSS, forming a robust crosslinked network. The crosslinking process was completed by heating the mixture to 120 °C. We also compared different crosslinking ratios to discuss the film performance. Lithium salts were incorporated to investigate the effect of varying lithium salt concentrations. According to EIS measurements, the best-performing system was crosslinked PVA-*g*-SBMA with PEDOT:PSS 0.1 wt% and LiTFSI 0.015 wt%, which reached conductivities of 4.9 × 10^−4^ S/cm at room temperature. We also explored the film’s thermal properties, morphologies, and chain interactions in this research.

## 1. Introduction

In modern energy storage devices, lithium-ion batteries (LIBs) are the mainstream choice, occupying a significant position in both automotive batteries and electronic devices [1]. LIBs are favored for their high energy density [2], extended cycle life, low self-discharge, rapid charging capabilities, and improved sustainability [3]. These characteristics make them well-suited for portable electronics and electric vehicles [4], contributing to a reduced footprint [5]. However, conventional liquid LIBs face challenges such as electrolyte leakage [6], limited thermal stability [7], and concerns stemming from their toxic electrolyte components [8]. These drawbacks reduce the battery’s performance [9]. As a result, many studies are now focusing on solid-state lithium-metal batteries (SSLMBs) [10], which use solid-state electrolytes (SSEs) to address the problems associated with traditional liquid electrolyte batteries [11]. Compared to liquid electrolytes, solid polymer electrolytes (SPEs) can effectively improve battery safety due to their higher thermal stability and lower combustion risk [12]. At the same time, SPEs typically exhibit lower ion conduction losses, which help to enhance the battery’s energy density and cycle life [13]. Furthermore, their advantages in operating temperature range and chemical stability enable them to adapt to diverse applications [14], including use in high-temperature and extreme environments [15], effectively improving non-flammability, safety, and processing performance [16]. In the SPE system, lithium ions dissolve in the polymer solvent and move through the amorphous and crystalline phases in the polymer structure [17]. In the amorphous region, lithium ions can migrate freely between the polymer’s free volume and polymer segments. However, since the amorphous region in polymers mainly depends on the properties of the polymer itself, the ability to improve conductivity is limited, which affects the performance of the battery.

In this study, the incorporation of the zwitterionic monomer SBMA ([2-(methacryloyloxy)ethyl]dimethyl-(3-sulfopropyl)ammonium hydroxide) into the polymer matrix serves a dual function: enhancing salt dissociation and regulating ionic selectivity [18]. Unlike conventional solid polymer electrolytes (SPEs), where both cations and anions possess comparable mobility [19], the zwitterionic nature of SBMA introduces a unique ‘isoelectric trap’ mechanism that fundamentally alters the ion transport dynamics.

The SBMA moiety contains both a cationic quaternary ammonium group and an anionic sulfonate group covalently tethered within the same side chain. Upon the addition of lithium salt (e.g., LiTFSI), the high dipole moment of the zwitterion effectively lowers the energy barrier for salt dissociation, increasing the concentration of mobile charge carriers [20]. Crucially, the cationic center of the SBMA interacts electrostatically with the free anions (TFSI^-^), effectively acting as an anion trap. This restriction of anionic motion mitigates the adverse effects of concentration polarization typically observed in dual-ion conductors [21,22].

Consequently, while the system remains a salt-in-polymer electrolyte, it exhibits transport behaviors analogous to Single-Ion Conducting Polymer Electrolytes (SICPEs). By immobilizing the counter-anions via the zwitterionic frame, as reported to increase Li^+^ selectivity in related zwitterionic polymer systems, often resulting in elevated Li^+^ transference numbers (tLi^+^ > 0.6), depending on the polymer architecture and salt content, this approach mimics the high-efficiency transport of SICPEs [21]. Thus, the SBMA-modified electrolyte functions as a ‘pseudo-single-ion conductor,’ bridging the gap between the high conductivity of traditional SPEs and the high selectivity of SICPEs.

Poly(vinyl alcohol) (PVA), on the other hand, is a biodegradable polymer with excellent physicochemical properties [23]. Its structure consists of hydroxyl groups attached to the carbon chain, which can form hydrogen bonds [24]. These hydrogen bonds help form polymer mixtures, giving PVA good solubility and film-forming properties [25]. PVA has a semicrystalline structure, primarily composed of crystalline and amorphous regions. In semicrystalline polymers, the ordered crystalline regions significantly enhance the material’s mechanical properties by creating localized zones of dense, highly organized molecular structure that improve overall strength, stiffness, and dimensional stability, making it suitable for applications in films, fibers, and composites [26]. More importantly, the hydroxyl functional groups in the PVA molecular structure give it excellent chemical modifiability. Through methods such as graft copolymerization and crosslinking modification, its solubility, mechanical properties, and biocompatibility can be further adjusted to meet different application needs.

Lithium-ion transport in polymer electrolytes is primarily facilitated by the material’s amorphous regions, where disordered and flexible polymer chain structures create optimal pathways for ion migration. These amorphous zones offer lower energy barriers and increased free volume, allowing lithium ions to move more freely compared to the tightly packed crystalline regions. Experimental studies show a sharp increase in ionic conductivity above the glass transition temperature (T_g_), confirming that Li^+^ mobility is closely tied to amorphous-phase dynamics [27,28]. This unique transport mechanism is critical for developing advanced energy storage technologies, particularly in next-generation solid-state batteries, where efficient ion conductivity can significantly enhance performance and reliability. Therefore, the semicrystalline nature of PVA can effectively create channels for ion movement, making it widely applicable in solid polymer electrolytes. However, the application of polymer materials is limited by their inherent mechanical properties, ionic transport capability, and electrochemical stability. Graft polymerization, as an effective polymer modification technique, can improve the structure and properties of materials by introducing functional side chains to the main polymer backbone. Graft polymerization strategies include free radical polymerization, atom transfer radical polymerization (ATRP), and reversible addition-fragmentation chain transfer (RAFT) polymerization [29]. The main chain, composed of polymers with a high T_g_, provides mechanical strength to the SPE, while the side chains, made from monomers with low T_g_ and high lithium salt solubility, provide high ionic conductivity. These methods allow the grafting of ion-conducting and ionophilic monomers onto the polymer backbone, effectively improving ionic transference numbers and interface compatibility [30].

Therefore, we designed a grafting strategy to attach [2-(methacryloyloxy)ethyl] dimethyl-(3-sulfopropyl)ammonium hydroxide (SBMA) to PVA. SBMA is a molecule with amphiphilic characteristics, containing both hydrophilic and hydrophobic groups in its structure [31]. This dual nature makes SBMA a versatile molecule with a wide range of applications. The hydrophilic part contains a sulfonic acid group (-SO_3_^−^) and a quaternary ammonium group (-N^+^(CH_3_)_2_), forming a zwitterionic structure [32]. Amphiphilic molecules exhibit strong intramolecular and intermolecular interactions, and when ions are added, these interactions are disrupted, allowing the chains to move freely within the electrolyte [33]. The SBMA chain also contains a methacryloyl group, which provides hydrophobicity [34], allowing it to remain stable in organic matrices, thereby enhancing the material’s structural integrity and durability. These characteristics make SBMA a promising component for improving the performance of SPEs. The zwitterionic structure can enhance ion transport capabilities by facilitating transitions between the ionic species while also maintaining the structural stability of the material. Min et al. synthesized a PVA-*g*-PSBMA zwitterionic graft copolymer to adjust PVA properties and increase ion transport. The results show high specific capacitance (46.6 F/g), energy density (3.67 Wh/kg), and power density (186 W/kg).

However, the primary limitation of polymers as electrolyte materials lies in their inherent stability challenges, which significantly compromise long-term performance and cycling durability in electrochemical applications. Mechanical stress caused by volume changes during prolonged charge and discharge cycles can lead to film cracking. Therefore, it is essential to enhance the mechanical properties of polymers. Crosslinking is an effective method for reducing crystallinity while significantly improving the mechanical strength and thermal stability of polymers [35]. Yu et al. utilized electrospinning to create a PEO/PEDOT:PSS/nanofiber structure for tumor cell signal detection. However, they faced challenges due to the poor wet stability of both PEDOT:PSS and the nanofiber mat. To resolve this issue, they crosslinked the structure, either self-crosslinked or with PEDOT:PSS, forming bonds between PSS and PEO, and added a crosslinking agent (GOPS). The epoxide groups in GOPS chemically reacted with excess PSS phosphate groups. The methoxy silane groups of GOPS molecules formed crosslinking bonds, which improved the stability and water resistance of the conductive material [36].

This research aims to utilize the excellent mechanical properties of PVA as the backbone to provide superior flexibility for solid-state electrolyte films while utilizing the amphiphilic structure of SBMA to create pathways for lithium salt transport. The positive lithium ions and the sulfonic groups (−SO_3_^−^) in SBMA will attract the lithium ions, facilitating their transport. The grafted structure will provide multiple sulfonic groups within the solid-state electrolyte to enhance the overall ion transport speed and improve conductivity. Free radical polymerization will be used to synthesize the grafted polymer PVA-*g*-SBMA. Furthermore, to enhance mechanical robustness and water resistance, PEDOT:PSS is introduced at an ultralow loading (0.1 wt%) primarily as a sulfonated crosslinkable component (PSS-rich phase) that participates in the dual crosslinking reaction with PVA and GOPS. At this loading, PEDOT does not form a continuous electronic percolation pathway, and the films remain electronically insulating while enabling ion transport. The sulfonic groups on PSS in PEDOT:PSS can crosslink with the hydroxyl groups of PVA at appropriate temperatures, while the epoxide groups on GOPS can form covalent bonds with the sulfonic groups of PSS during heating. Lithium ions primarily move through the sulfonic groups in SBMA. After crosslinking, the resulting stable structure enables the preparation of high-strength, high-conductivity solid-state electrolyte films for solid-state devices. Therefore, we investigate how varying lithium salt (LiTFSI) concentrations influence the surface, thermal, and conductive properties of the crosslinked PVA-*g*-SBMA films and also determine the optimal ratio for achieving the highest ionic conductivity by adjusting the concentrations of PEDOT:PSS and GOPS in the crosslinking process.

## 2. Materials and Methods

### 2.1. Materials

Poly(vinyl alcohol) (PVA, Mw = 6000, 98%) from Polysciences, [2-(methacryloyloxy)ethyl]dimethyl-(3-sulfopropyl) ammonium hydroxide (SBMA, 98%) from TCI America, Cerium(IV) ammonium nitrate (CAN, 99%), 2,2-azobis(2-methylpropionitrile) (AIBN, 99%), poly(3,4-ethylenedioxythiophene):poly(styrenesulfonate) (PEDOT:PSS, 1.3 wt%in H_2_O), (3-glycidyloxypropyl)trimethoxysilane (GOPS, 99%), and bis(trifluoromethane)sulfonimide lithium salt (LiTFSI, ≥99%) were purchased from Sigma Aldrich MN, America. Dimethyl sulfoxide (DMSO), ethyl acetate, and n-hexane were purchased from Merck, MA, America. All the chemical materials and solvents were used as received without any modification or purification.

### 2.2. Synthesis PVA-g-SBMA Graft Copolymer

The free radical polymerization began by adding PVA (5.0 g) and SBMA (3.33 g) into a reaction flask, then dissolving them in DMSO (95 mL). The mixture was homogenized at 65 °C under a nitrogen atmosphere, and CAN (250 mg) as an initiator dissolved in DMSO (5 mL) was subsequently added to the reaction flask to start the reaction for 18 h. CAN generated Ce^4+^ ions, which oxidized the hydroxyl groups of PVA to form alkoxy radicals. These radicals initiated graft polymerization by reacting with the vinyl group of SBMA. After complete reaction, AIBN (250 mg) as a terminator dissolved in DMSO (5 mL) was added and reacted for 8 h. The resulting product was then poured into hexane: ethyl acetate (*v*:*v* = 1:1) and vigorously stirred for one day to dissolve impurities. This step was repeated once to ensure product purity. The product dissolved in ethyl acetate was then purified using a Rota Vapor to remove hexane and ethyl acetate from the solution. Finally, the product was further purified using dialysis bags, soaking in deionized water with continuous agitation for five days to enhance purification. The dialyzed product was then freeze-dried to obtain powdered graft polymer.

### 2.3. Crosslinking Reaction

The synthesized PVA-*g*-SBMA powder was further combined with PEDOT:PSS using GOPS as a crosslinking agent. First, the hydroxyl groups of PVA can crosslink with the sulfonic acid groups of PSS at an appropriate temperature. Second, the epoxy groups of GOPS are highly reactive and can undergo ring-opening reactions with the sulfonic acid groups of PSS upon heating. Lastly, crosslinking in this system can occur both between PVA and PSS and between GOPS and PSS. Through vigorous stirring, the components are uniformly mixed to form a network, which effectively enhances the mechanical properties of the polymer solid-state electrolyte film.

### 2.4. Fabrication of Solid Polymer Electrolyte

After completing the crosslinking reaction, 0.015 and 0.02 wt% lithium salt were added to the solution and vigorously stirred for 1 h to obtain a homogeneous mixture containing the lithium salt. Bis(trifluoromethane)sulfonimide lithium salt (LiTFSI) was selected as the lithium source. The resulting solution was then placed in a vacuum oven and dried at 80 °C for 3 days to remove residual DMSO.

### 2.5. Properties Measurement

The structure of the graft copolymers was characterized by NMR ^1^H spectroscopy using a 500 MHz spectrometer (AVANCE III HD 400, Bruker, USA) with D_2_O as the solvent. Mn and PDI were analyzed by aqueous GPC (WAT011525 Pump and 2414 RI Detector, Waters, USA), detecting molecular weight range: 1000 to 80,000. FT-IR spectra were recorded using an FT/IR-6700 type A spectrometer (JASCO, Japan) with a ZnSe ATR crystal to confirm the functional groups in the polymer structure.

The surface morphology of crosslinked films was observed by SEM (JSM-7800F, JEOL, Japan). Surface behavior was confirmed by contact angle measurement (Model 100SB, Sindatek, Taiwan). The thermal properties were measured by DSC (DSC 4000, PerkinElmer, USA) to analyze the melting and crystallization behavior of the films with a heating and cooling rate of 10 °C/min from 40 to 200 °C. The thermal decomposition behavior was evaluated by TGA (Q500, TA Instruments, USA) under a nitrogen atmosphere with a heating rate of 10 °C/min from 40 to 600 °C. The mechanical properties were measured by DMA (Q800, TA Instruments, USA) with a heating rate of 2 °C/min, frequency at 0.1 Hz, and amplitude of 2 µm after being heated to 100 °C for 10 min from −50 to ~80 °C. Polarized optical microscopy (BX53, OLYMPUS, Japan) was employed to study the crystallization behavior of the polymer. The sample was drop-cast on a cover glass, dried in a vacuum oven at 80 °C, and then heated at a rate of 10 °C/min.

The ionic conductivity of the polymer electrolyte films was determined using electrochemical impedance spectroscopy (EIS), measured with a potentiostat (PGSTAT128N, Metrohm Autolab, The Netherlands) in a two-electrode configuration with blocking electrodes to suppress faradaic contributions. The film sample was placed between a clamp with a contact radius of 0.5 cm. Impedance measurement was conducted in the frequency range of 0.1 to 500,000 Hz with an applied AC voltage of 5 mV. The ionic conductivity (σ, S/cm) was calculated using the following equation:

$$\sigma =\frac{L}{R\times A}$$

where L is the film thickness, R is the resistance, and A is the contact area.

## 3. Results and Discussion

### 3.1. Synthesis and Characterization of PVA-g-SBMA

**Figure 1:**
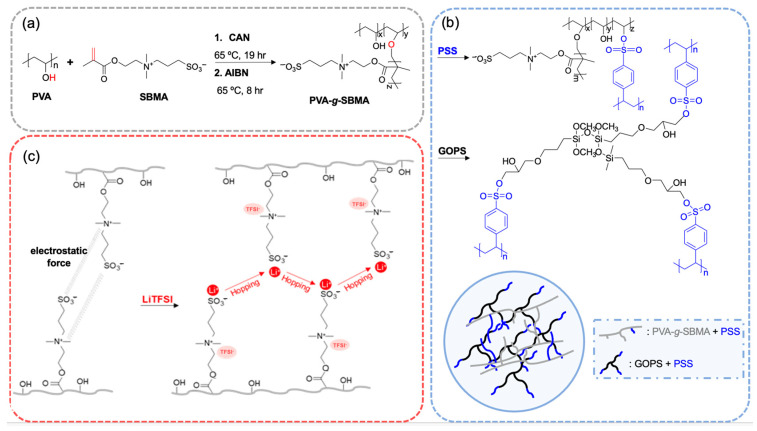
(**a**) PVA-g-SBMA synthesis reaction, (**b**) crosslinking mechanism, and (**c**) migration of lithium ions in solid polymer electrolyte.

The synthesis mechanism is shown in Figure 1a. CAN serves as an initiator to start the free radical polymerization due to its strong oxidizing capability. It activates hydroxyl groups (–OH) in PVA to start graft polymerization. During the reaction, CAN decomposes to produce nitrate ions (NO_3_^−^) and cerium (IV) ions (Ce^4+^). Ce^4+^ oxidizes the hydroxyl groups in PVA, generating alkoxy radicals (R−O •), as shown below:

R−OH + Ce^4+^ → R−O • + Ce^3+^ + H^+^

These radicals serve as initiation sites for graft polymerization. The C=C bond in SBMA reacts with the alkoxy radicals on PVA, forming new radicals and enabling grafting, as shown below:

R−O • + CH_2_=C(CH_3_)COO(CH_2_)_3_−SO_3_−N^+^(CH_3_)_3_

→ R−O−CH_2_−C • (CH_3_)−COO(CH_2_)_3_−SO_3_−N^+^(CH_3_)_3_

This reaction opens the double bond and attaches SBMA onto the PVA chain. After grafting, AIBN is added as a terminator. Upon heating, AIBN generates stable radicals that couple with the remaining active radicals in the system, thereby terminating the polymerization. Then, the crosslinking mechanism is shown in Figure 1b. Hydroxyl groups on PVA are crosslinked with sulfonic acid groups on PSS, and GOPS are bonded with PSS via epoxy–sulfonic acid reactions. Dual crosslinking between PVA–PSS and GOPS–PSS resulted in a network, improving the film’s mechanical strength. Lithium salt was mixed into films to form the solid polymer electrolyte, which is shown in Figure 1c. DMSO is selected as the solvent based on Tables S1 and S2. Films prepared with DI water exhibited reduced flexibility and were adversely affected by lithium-ion migration. Therefore, choosing DMSO as the solvent in this system is preferred.

The proton assignment of PVA-*g*-SBMA is investigated from the ^1^H NMR spectrum. Figure S1 shows the D_2_O solvent peak appearing at 4.8 ppm, and residual DMSO signals appear at 2.1 and 2.7 ppm. Analysis of the spectrum reveals that peaks labeled a, b, c, and d correspond to protons from PVA, while peaks labeled e to m are attributed to SBMA side chains. For comparison, pure SBMA monomer exhibits characteristic peaks at 2.3, 3.2, 3.6, 3.8, 4.7, and 5.8 ppm, with the peak at 5.8 ppm corresponding to the vinyl protons of the C=C double bond. No significant signals are detected in the 5.5–6.0 ppm region, indicating that the C=C double bonds of SBMA participate in the radical-initiated grafting reaction and are consumed during polymerization. The ^1^H NMR spectrum of pure PVA also shows signals for the hydroxyl protons at 1.6–1.8 ppm and methine protons adjacent to the hydroxyl groups at approximately 4.1 ppm. Additional peaks in the 3.5–4.0 ppm region correspond to the SBMA side chains. The absence of these peaks in the pure PVA spectrum confirms the successful grafting of SBMA onto the PVA.

Table S3 shows the results from GPC. The PVA-*g*-SBMA graft copolymer has Mn: 15,755 g/mol and PDI: 1.17. We further discuss the grafting ratio. Assume that each SBMA unit grafted onto the PVA exists as a single monomer. The grafting degree is calculated to be approximately 25.7%.

### 3.2. Characterization of Crosslinked PVA-g-SBMA Films

**Figure 2:**
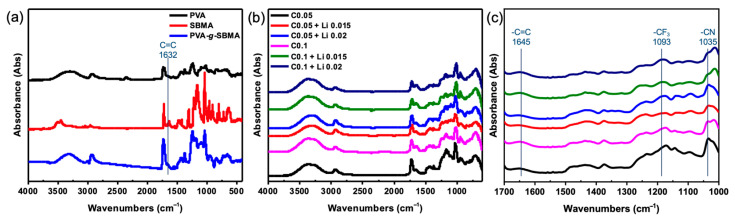
FT-IR spectra of (**a**) PVA-*g*-SBMA w/wo crosslink, (**b**) crosslinked films with different lithium ratios, and (**c**) detail scale showing peaks between 1700 and 1000 cm^−1^.

The crosslinking ratio is selected as 0.05 and 0.1 wt% (Table S4). At the lowest concentration of 0.01 wt%, the insufficient crosslinking prevented the formation of a cohesive film. This led to incomplete casting and delamination upon release from the mold. In contrast, films prepared at higher concentrations (0.05 wt% and 0.1 wt%) could be cleanly detached from the mold, maintaining structural integrity and exhibiting good mechanical properties. We next characterize the crosslinked PVA-*g*-SBMA films. The grafting reaction is initiated via a free-radical polymerization, in which the potent oxidizing agent, ceric ammonium nitrate (CAN), oxidizes the hydroxyl groups (–OH) on the PVA backbone to form alkoxy radicals. These radicals then react with the vinyl groups (C=C) in SBMA, forming grafted structures. Upon successful grafting, the C=C stretching vibration of SBMA should no longer be observed in the final product. Therefore, as shown in Figure 2a, the FTIR spectrum of PVA displays a broad O–H stretching band at 3300 cm^−1^ and a C–H stretching band at 2930 cm^−1^. The SBMA monomer shows a C=C stretching peak at 1632 cm^−1^ and a C–N stretching band at 1035 cm^−1^. In contrast, the PVA-*g*-SBMA spectrum retains the C–N stretching peak at 1035 cm^−1^ and the C–H stretching around 2900 cm^−1^, while the C=C peak at 1632 cm^−1^ disappears. This indicates that the double bond of SBMA was successfully consumed, confirming the successful grafting of SBMA onto the PVA.

Figure 2b further compares the FTIR spectra before and after crosslinking. The broad O–H stretching band around 3400 cm^−1^ and the C–H stretching peak at 2900 cm^−1^ are observed from PVA in the crosslinked sample. The characteristic C–N stretching band at 1035 cm^−1^ is present from SBMA. C=C vibration peaks at 1645 and 711 cm^−1^ from PEDOT:PSS confirm its incorporation into the polymer matrix. In addition, the intensity of the O–H stretching band around 3400 cm^−1^ and the C–O–C stretching peak at 1096 cm^−1^ increases. These enhancements are attributed to the ring-opening reaction between the epoxy group of GOPS and the hydroxyl group of PSS during the crosslinking process. Therefore, successful crosslinking can be inferred from the increased intensity of C–O–C stretching and the associated O–H signals. When comparing different crosslinking concentrations, 0.05 wt% (C0.05) and 0.1 wt% (C0.1), the peaks at 1645 cm^−1^ (C=C from PEDOT:PSS) and 1035 cm^−1^ (C–N from SBMA) show increased intensity at a higher crosslinking ratio, suggesting higher bonding of PEDOT:PSS with the graft copolymer. The higher SBMA peak may result from a denser polymer network structure due to an increased crosslinking ratio. Meanwhile, the O–H stretching intensity in the C0.1 sample slightly decreases compared to C0.05, possibly due to greater consumption of free hydroxyl groups in the crosslinking reaction. Upon the addition of lithium salt (LiTFSI), a new absorption peak appears at 1193 cm^−1^, corresponding to the –CF_3_ stretching vibration of LiTFSI, indicating successful incorporation of the salt into the polymer film. Moreover, the intensities of the C=C stretching at 1645 cm^−1^ and the C–N stretching at 1035 cm^−1^ also change upon lithium salt doping, as shown in Figure 2c. These results suggest that a higher crosslinking degree (C0.1) improves the structural stability, and the presence of lithium salt interacts with functional groups from SBMA, leading to higher peak values in their corresponding FTIR signals.

### 3.3. Surface Morphology of Crosslinked PVA-g-SBMA Films

**Figure 3:**
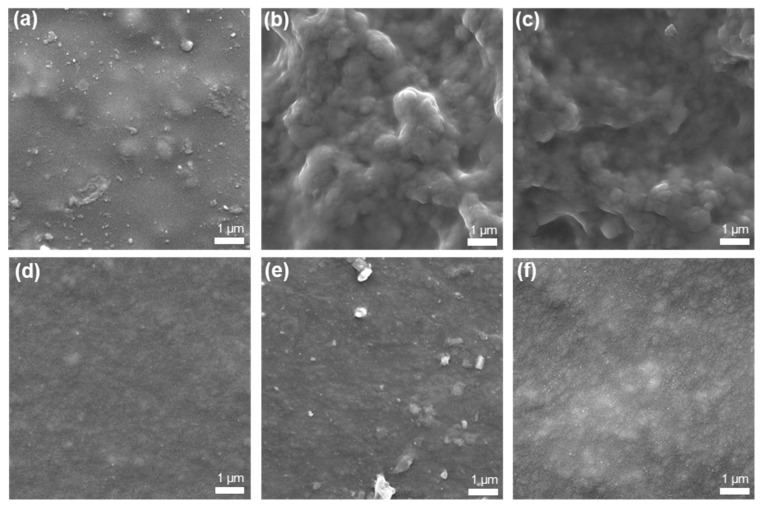
SEM images of films (**a**) C0.05, (**b**) C0.05 + LiTFSI 0.015 wt%, (**c**) C0.05 + LiTFSI 0.02 wt%, (**d**) C0.1, (**e**) C0.1 + LiTFSI 0.015 wt%, and (**f**) C0.1 + LiTFSI 0.02 wt%.

Figure 3 illustrates the surface morphology of crosslinked PEDOT:PSS films at two crosslinking ratios: C0.05 and C0.1. In the C0.05 system, the surface without LiTFSI (Figure 3a) exhibits slight protrusions. As LiTFSI concentration increases to 0.015 wt% (Figure 3b), the surface becomes rougher. Further increasing the concentration (Figure 3c) maintains this roughness. However, in the C0.1 system, the surface without LiTFSI (Figure 3d) is smooth, and protrusions are hard to find. Minor surface irregularities appear after adding 0.015 wt% LiTFSI (Figure 3e), but after adding higher concentrations (Figure 3f), the surface remains relatively smooth. These observations suggest that a higher concentration of crosslinking agent (C0.1) is beneficial to the film formation. They tend to have smooth and uniform surfaces during film fabrication. In contrast, films with a lower concentration of crosslinking agent (C0.05) are more difficult to maintain with surface uniformity.

We also investigate the changes in surface hydrophilicity after crosslinking resulting from the amphiphilic property of SBMA. As shown in Table S5, the contact angle of C0.05 sample without LiTFSI is 103.72°, exhibiting a relatively high contact angle, which indicates lower hydrophilicity. At a concentration of 0.015 wt% LiTFSI, the contact angle decreases to 101.33°. This decrease is likely a result of lithium-ion interactions with SBMA, which enhance hydrogen bonding involving the sulfonate groups (–SO_3_^−^). Increasing the LiTFSI concentration to 0.02 wt% further lowers the contact angle to 89.61°, suggesting a greater exposure of hydrophilic groups on the surface. At a higher PEDOT:PSS ratio, the contact angle of C0.1 without LiTFSI is 67.59°, which shows improved hydrophilicity compared to C0.05, likely due to the polar sulfonic acid groups (–SO_3_H) in PEDOT:PSS. Increasing LiTFSI to 0.015 wt% causes minimal change. The contact angle decreases to 61.26°. While 0.02 wt% maintains low contact angles to 43.21°, indicating a stable hydrophilic surface. These results suggest that crosslinked films retain good hydrophilicity, with the lithium salt serving to further modulate surface properties.

### 3.4. Thermal Behavior of Crosslinked PVA-g-SBMA Films

**Figure 4:**
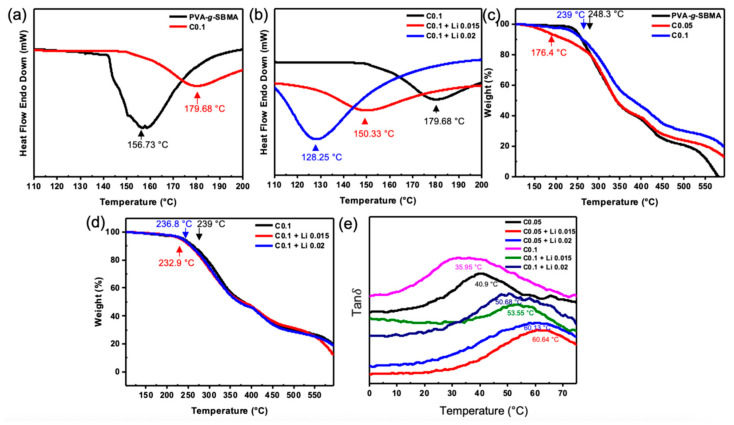
DSC curves with heating rate 10 °C/min (**a**) PVA-*g*-SBMA w/wo crosslink, (**b**) C0.1 with different LITFSI ratio. TGA curves with heating rate 10 °C/min in N2 (**c**) PVA-*g*-SBMA w/wo crosslink, (**d**) C0.1 with different LiTFSI ratio, and (**e**) tan delta curves from DMA with heating rate 2 °C/min in N2 after heated 100 °C 10 min; frequency: 0.1 Hz; amplitude: 2 µm.

DSC analysis is conducted to investigate the thermal properties of PVA-*g*-SBMA films before and after crosslinking. From Figure 4a, the non-crosslinked sample shows a Tm of 156.73 °C and a ΔHm of 254.75 J/g, indicating a relatively low melting point and high crystallinity. Upon crosslinking (C0.1), the Tm rises to 179.68 °C, while the ΔHm significantly decreases to 88.44 J/g. These changes suggest that crosslinking effectively alters the polymer structure. The elevation in melting temperature can be attributed to restricted polymer chain mobility due to crosslinking, enhancing intermolecular interactions, and thermal stability. Conversely, the reduction in enthalpy of fusion implies that crosslinking disrupts the crystalline regions of PVA, leading to decreased crystallinity.

To further investigate the influence of lithium salt incorporation on the thermal behavior of crosslinked PVA-*g*-SBMA films, the crosslinking ratio is fixed (C0.1), and various concentrations of LiTFSI are added. From Figure 4b, the C0.1 without LiTFSI exhibits a Tm of 179.68 °C and a ΔHm of 88.44 J/g, indicating relatively high thermal stability and low crystallinity.

Upon adding LiTFSI, there is a decreasing trend in Tm with higher lithium salt concentration. After adding 0.015 wt% LiTFSI, Tm decreased to 150.33 °C and 128.25 °C at 0.02 wt% LiTFSI. This reduction in Tm indicates that the addition of lithium salt compromises the thermal stability. The insertion or disruption of lithium ions in the crystalline regions leads to less ordered crystal packing and smaller crystallite sizes and reduced structural order. The consequent lattice destabilization facilitates thermal disordering, thereby lowering the energy required for phase transition. However, in terms of ΔHm, a significant increase is observed with higher LiTFSI concentrations. The ΔHm rises from 88.44 J/g to 112.82 J/g with the addition of 0.015 wt% LiTFSI and further increases to 348.36 J/g at 0.02 wt% LiTFSI. This increase may be attributed to the stronger interactions between sulfonic acid groups and the positively charged lithium ions. The elevated ΔHm suggests that more thermal energy is required to dissociate these interactions, thereby resulting in a higher overall ΔHm.

The thermal stability of the film is crucial for solid-state electrolyte applications. TGA is conducted to investigate thermal degradation further. As shown in Figure 4c, PVA-g-SBMA without crosslinking exhibits a T-5 of 248.3 °C, indicating high initial thermal stability. At a 0.05 crosslinking ratio, T-5 drops significantly to 176.4 °C, indicating thermal stability at lower crosslinking levels. However, increasing the crosslinking ratio to 0.1 raises T-5 to 239 °C, close to that of the uncrosslinked sample. This indicates that higher crosslinking enhances the thermal stability of the solid-state electrolyte films.

To further discuss the effect of LiTFSI on thermal stability in crosslinking films, the TGA curves are shown in Figure 4d. The T-5 of C0.1 without LiTFSI is 239 °C. When 0.015 wt% LiTFSI is added, T-5 decreases to 232.9 °C. With further addition of LiTFSI to 0.02 wt%, T-5 slightly increases to 236.8 °C. Although these values are slightly lower than those of the salt-free film, the overall reduction in thermal stability is minimal. This suggests that while LiTFSI incorporation at this crosslinking density mildly reduces initial thermal stability, the impact is not significant.

The effect of varying LiTFSI doping concentrations on the mechanical properties and thermal transition behavior of the films was studied by DMA in this study. As shown in Figure 4e, the curves represent the Tan δ of the films with the peak corresponding to Tg. For the C0.1 without LiTFSI, Tg is 35.95 °C. Upon adding 0.015 wt% LiTFSI, Tg significantly increases to 53.55 °C. When the concentration is further increased to 0.02 wt%, Tg slightly decreases to 50.68 °C. This suggests that moderate LiTFSI doping can enhance thermal stability and restrict chain mobility. For the lower crosslinking density (C0.05), the C0.05 without LiTFSI shows Tg of 40.9 °C. With 0.015 wt% and 0.02 wt% LiTFSI, Tg increases to 60.64 °C and 60.13 °C, respectively. The marked rise in Tg indicates that LiTFSI addition effectively improves thermal stability. In summary, adding LiTFSI yields higher Tg values compared to undoped films. Additionally, lower Tg observed in highly crosslinked samples (C0.1) may offer better processability in subsequent applications.

To investigate the crystallization behavior of the films, Figure S2a presents the polarized optical microscopy images of the crosslinked film during the heating process from 40 °C to 250 °C. The film exhibits visible displacement, attributed to its increasing fluidity at elevated temperatures. However, even at temperatures approaching the thermal decomposition point, no crystal melting is observed. Similarly, Figure S2b depicts the cooling process from 250 °C back to 40 °C. No evidence of recrystallization is observed during this cooling cycle. Figures S3 and S4 correspond to films incorporated with 0.015 wt% and 0.02 wt% LiTFSI, respectively. Under identical thermal cycling conditions, both samples demonstrate comparable behavior. No discernible signs of crystallization, melting, or recrystallization are observed through polarized optical microscopy analysis. These observations indicate that the C0.1 system remains highly amorphous under the examined conditions. Furthermore, variations in lithium salt concentration within the tested range do not induce notable changes in crystallization behavior. These findings are in good agreement with the results obtained from DSC, which similarly show no detectable crystalline transitions.

To further investigate the internal molecular arrangement of the polymer, XRD analysis was conducted. As shown in Figure S5a, PVA (black curve) exhibits a diffraction peak at 2θ = 19.5°, corresponding to the crystalline regions characteristic of the semi-crystalline nature of PVA. This peak reflects the regular arrangement of the polymer main chains. After grafting with SBMA, the PVA-*g*-SBMA film (red curve), as well as the C0.1 crosslinked film (blue curve), exhibits slightly reduced peak intensity, while the peak position remains essentially unchanged. Therefore, based on the XRD results, no significant enhancement in amorphous characteristics can be confirmed. In Figure S5b, which focuses on the C0.1 system with varying LiTFSI concentrations, the C0.1 (black curve) represents the crosslinked sample without lithium salt, showing its primary diffraction peak at 2θ = 19.5°. Upon the addition of LiTFSI at concentrations of 0.015 wt% (red curve) and 0.02 wt% (blue curve), a slight increase in peak intensity is observed. However, the overall diffraction patterns do not exhibit substantial differences, and no clear evidence of crystallization can be deduced from the XRD data. The minimal intensity variations across different LiTFSI concentrations suggest that the presence of lithium salt does not significantly influence the crystalline structure of the films under the examined conditions.

### 3.5. Conductivity of Crosslinked PVA-g-SBMA Films

Because PEDOT:PSS is used at ultralow loading (0.1 wt%) and primarily participates in crosslinking, the films do not exhibit continuous electronic percolation; therefore, the conductivity values discussed below are attributed to ionic transport and evaluated from EIS under blocking-electrode conditions. EIS was used to study the ionic transport properties of polymer composite films with varying concentrations of LiTFSI (0–0.05 wt%) and different cross-linking factors (C0.1 and C0.05). The ionic conductivity was analyzed in the frequency domain, with data plotted as log ionic conductivity (S⋅cm^−1^) versus log frequency (Hz). Presenting EIS data in conductivity–frequency coordinates provides several benefits for polymer electrolytes. First, the low-frequency plateau region (left side of the plot) corresponds directly to the long-range (DC) ionic conductivity, which is the relevant transport property for battery operation. The crossover frequency (ωc), where the curve departs from the plateau into a frequency-dependent dispersive region, reflects the characteristic relaxation time (τ = 1/ωc) of mobile ions. Finally, the high-frequency dispersive regime follows a power-law dependence (σ ∝ ω^s), which yields mechanistic insight into ion hopping and segmental dynamics. This representation, therefore, highlights both absolute conductivity values and the dynamic processes governing ion motion, while suppressing the overlap of multiple processes often observed in Nyquist plots.

To investigate the influence of lithium salt concentration on the conductivity performance of the films, we first conduct the C0.1 system with varying concentrations of LiTFSI (0–0.05 wt%), which previously demonstrated superior thermal and mechanical properties.

**Figure 5:**
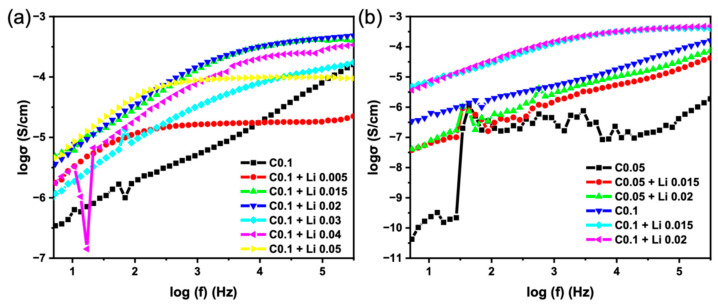
Frequency-dependent ionic conductivity of polymer composite electrolytes. (**a**) C0.1 system with varying LiTFSI content, showing increased DC conductivity and faster relaxation with higher salt ratios. (**b**) Comparison of C0.1 and C0.05 systems, highlighting the superior conductivity and stability of the C0.1 network.

The effect of LiTFSI incorporation in C0.1 films. Figure 5a shows the ionic conductivity spectra of the C0.1 system with different LiTFSI salt ratios. The pristine C0.1 sample exhibits the lowest conductivity (5.5 × 10^−5^ S/cm) (black line), except for the sample 0.005 wt% LiTFSI (red line), which significantly enhances the ionic conductivity across all frequencies. With increasing salt content, two trends are evident: (i) the low-frequency plateau conductivity (σdc) rises by nearly an order of magnitude, indicating an increased concentration of mobile Li^+^ carriers, where the conductivity is independent of frequency. This is the regime of long-range ionic transport, which is the most desirable property for a solid electrolyte and (ii) the crossover frequency shifts to higher values, implying faster ionic relaxation and shorter hopping times. The slope of the dispersive region also changes slightly with salt loading, which suggests a modification of the ion–polymer interaction environment and possibly reduced correlation in Li^+^ motion.

Optimum LiTFSI Ratio: The sample with the highest plateau, and therefore the highest DC ionic conductivity, is C0.1 + Li 0.015 and C0.1 + Li 0.02 (4.9 × 10^−4^ S/cm (green line) 4.6 × 10^−4^ S/cm (blue line), respectively). Adding too much or too little LiTFSI salt decreases the overall conductivity. This is a common phenomenon in solid polymer electrolytes, where an optimal concentration of salt provides enough mobile charge carriers (ions) without causing excessive ion aggregation, which would hinder their movement.

AC Conductivity Dispersion: At higher frequencies (right side of the plot), the conductivity for all samples increases significantly. This is the AC conductivity regime, where the conductivity becomes frequency-dependent. This behavior is due to localized, short-range hopping of ions that cannot complete long-range transport within the short time frame of the high-frequency alternating electric field.

Effect of LiTFSI Concentration: As the LiTFSI concentration changes, both the DC and AC conductivity are affected. The optimal concentration of 0.015−0.02% LiTFSI results in the highest DC conductivity, indicating the best balance between carrier concentration and mobility.

Next, we aimed to compare the influence of different crosslinking densities on the conductivity performance. C0.05 and C0.1 were investigated with varying LiTFSI concentrations (0, 0.015, and 0.02 wt%) to assess their conductivity behavior. The conductivity of the C0.05 sample is significantly lower than that of C0.1 (2 × 10^−7^ S/cm) (black line), even with salt addition. In particular, the pristine C0.05 curve approaches the instrumental noise floor at low frequencies, with irregular fluctuations indicative of electrode polarization and poor percolation pathways for ion transport. Upon salt addition, conductivity increases significantly: 0.015 wt% LiTFSI to the C0.05 system (red line), conductivity increases to 1.1 × 10^−5^ S/cm; however, the improvement remains less pronounced than in C0.1. For many of the C0.05 samples, especially those with low LiTFSI concentrations, there is not a clear, flat low-frequency plateau. The conductivity continues to decrease at lower frequencies. This indicates that the long-range ionic transport is very limited in these materials, and the measured conductivity is dominated by localized AC processes. Compared to the C0.1 system with the same LiTFSI concentration (light blue line), the C0.1 film exhibits conductivity one order of magnitude higher. This suggests that while reduced cross-linking increases segmental flexibility, it does not necessarily promote efficient ionic conduction. Instead, a higher degree of cross-linking (C0.1) appears to provide a more robust ionic pathway network and better salt dissociation environment, leading to superior conductivity.

The combined results reveal that both structural factors (cross-linking density) and compositional factors (salt concentration) strongly influence ionic transport. The high cross-linking system (C0.1) offers enhanced conductivity, likely due to a more interconnected polymer framework that stabilizes salt dissociation and provides well-defined ion-conducting channels. In contrast, the lower cross-linking system (C0.05) suffers from insufficiently connected pathways, which limit long-range transport despite higher polymer flexibility. The conductivity–frequency analysis further indicates that the C0.1 system not only achieves higher σdc values but also exhibits faster ion dynamics, as reflected by the shift in ωc to higher frequencies.

## 4. Conclusions

In summary, we successfully synthesized the graft copolymer PVA-*g*-SBMA via free radical polymerization, with structural confirmation provided by FTIR, GPC, and ^1^H NMR analyses. To address insufficient mechanical strength, crosslinking with PEDOT:PSS and GOPS was introduced, resulting in structurally stable solid-state electrolyte films. The highly crosslinked system (C0.1) exhibited a denser microstructure, enhanced thermal stability, and improved processability, as demonstrated by SEM, DSC, TGA, and DMA measurements.

The crosslinked PVA-g-SBMA films represent a high-performance alternative to traditional all-solid-state materials. For example, unlike toxic, non-flexible, unstable (in air and moisture environments), and expensive sulfide-based solid electrolytes, or heavy and brittle oxides, this polymer system offers a lightweight, cost-effective solution with superior ionic conductivity, mechanical integrity, and chain flexibility.

A key distinction of this material is its versatility: it is engineered to function not just as a standard polymer separator but also as a catholyte within the electrode to facilitate seamless ion transport. This is achieved through a precise material architecture:

Optimized Ionic Flow: Incorporation of LiTFSI (0.015–0.02 wt%) ensures high ionic conductivity.

Structural Synergy: A low loading of PEDOT:PSS (0.1 wt%) reacts with the GOPS crosslinker and PVA hydroxyl groups to form a robust network.

Collectively, these attributes position the PVA-g-SBMA system as a scalable, robust, and high-performance electrolyte for next-generation safe electrochemical applications.

This work focuses on establishing a synthesis and crosslinking strategy to achieve mechanically robust zwitterionic SPE films and elucidating their structure–property–transport relationships. Future studies will evaluate the Li^+^ transference number, interfacial stability against lithium metal (stripping/plating), and full-cell performance to further validate the electrolyte under practical battery conditions.

## Data Availability

The original contributions presented in this study are included in the article and Supplementary Materials. Further inquiries can be directed to the corresponding authors.

## Supplementary Materials

The following supporting information can be downloaded at: https://www.mdpi.com/article/10.3390/polym18030343/s1, Table S1. Solubility of PVA-g-SBMA in different solvent. Table S2. Mn, Mw, and PDI results from GPC. Table S3. Morphology and flexibility of PVA-g-SBMA films in different solvent. Table S4. Morphology of different crosslinking ratio PVA-g-SBMA films. Table S5. Different crosslinking ratio with LiTFSI contact angle results. Figure S1. Protons assignment of PVA-g-SBMA and ^1^H NMR spectrum of PVA-g-SBMA. Figure S2. Polarized Optical Microscopy images of the C0.1 film under a heating and cooling rate of 10 °C/min: (a) during heating and (b) during cooling. Figure S3. Polarized Optical Microscopy images of the C0.1 + LiTFSI 0.015 wt% film under a heating and cooling rate of 10 °C/min: (a) during heating and (b) during cooling. Figure S4. Polarized Optical Microscopy images of the C0.1 + LiTFSI 0.02 wt% film under a heating and cooling rate of 10 °C/min: (a) during heating and (b) during cooling. Figure S5. XRD patterns of (a) PVA, PVA-g-SBMA, and C0.1, and (b) the C0.1 system films. Figure S6. XPS spectra of PVA-g-SBMA and C0.1 film samples, with high-resolution core-level scans of C 1s, N 1s, O 1s, and S 2p.

## Abbreviations

PVA	Poly(vinyl alcohol)
SBMA	[2-(methacryloyloxy)ethyl]dimethyl-(3-sulfopropyl)ammonium hydroxide
PEDOT:PSS	Poly(3,4-ethylenedioxythiophene):poly(styrene sulfonate)
CAN	Cerium(IV) ammonium nitrate
AIBN	2,2-Azobis(2-methylpropionitrile)
GOPS	3-glycidyloxypropyl)trimethoxysilane
LiTFSI	Bis(trifluoromethane)sulfonimide lithium salt
DMSO	Dimethyl sulfoxide
NMR	Nuclear Magnetic Resonance
GPC	Gel Permeation Chromatography
FT-IR	Fourier-Transform Infrared spectroscopy
SEM	Scanning Electron Microscope
DMA	Dynamic Mechanical Analyzer
DSC	Differential Scanning Calorimetry
TGA	Thermogravimetric Analysis
EIS	Electrochemical Impedance Spectroscopy
