# Supplementary material for: Crosslinked Zwitterionic PVA-g-SBMA/PEDOT:PSS Networks for Mechanically Robust All-Solid-State Electrolytes
Source: Polymers (Basel). 2026 Jan 28;18(3):343. doi: 10.3390/polym18030343 (PMC12899714; doi:10.3390/polym18030343)
Supplement: Supplementary file 1 [file polymers-18-00343-s001.zip › polymers-4068943-supplementary.pdf]

Supporting Information

Crosslinked Zwitterionic PVA-g-SBMA/PEDOT:PSS Networks for Mechanically Robust All-Solid-State Electrolytes

Chia-Wen Wei [1], Chia-Yu Chen [1], Shyh-Chyang Luo [1], Dmitry G. Belov [2,*] and Szu-Nan Yang [3,*]

[1] Department of Materials Science and Engineering, National Taiwan University, No. 1, Sec. 4, Roosevelt Road, Taipei 10617, Taiwan; tiffanywei0305@gmail.com (C.-W.W.); osukon8@gmail.com (C.-Y.C.); shyhchyang@ntu.edu.tw (S.-C.L.)

[2] Prologium Innovation Europe, R&D Center, 6 Rue de la Terre de Fue, 91940 Les Ulis, France

[3] Prologium Technology Co., Ltd., No. 6-1, Ziqiang 7th Rd., Zhongli Dist., Taoyuan City 320, Taiwan

* Correspondence: dmitry@prologium.com (D.G.B.); vincent@prologium.com (S.-N.Y.)

Table of Contents

Table S1. Solubility of PVA-*g*-SBMA in different solvent.

Table S2. Mn, Mw, and PDI results from GPC.

Table S3. Morphology and flexibility of PVA-*g*-SBMA films in different solvent.

Table S4. Morphology of different crosslinking ratio PVA-*g*-SBMA films.

Table S5. Different crosslinking ratio with LiTFSI contact angle results.

Figure S1. Protons assignment of PVA-*g*-SBMA and ^{1}H NMR spectrum of PVA-*g*-SBMA.

Figure S2. Polarized Optical Microscopy images of the C0.1 film under a heating and cooling rate of 10 °C/min: (a) during heating and (b) during cooling.

Figure S3. Polarized Optical Microscopy images of the C0.1 + LiTFSI 0.015 wt% film under a heating and cooling rate of 10 °C/min: (a) during heating and (b) during cooling.

Figure S4. Polarized Optical Microscopy images of the C0.1 + LiTFSI 0.02 wt% film under a heating and cooling rate of 10 °C/min: (a) during heating and (b) during cooling.

Figure S5. XRD patterns of (a) PVA, PVA-*g*-SBMA, and C0.1, and (b) the C0.1 system films.

Figure S6. XPS spectra of PVA-*g*-SBMA and C0.1 film samples, with high-resolution core-level scans of C 1s, N 1s, O 1s, and S 2p.

Table S1. Solubility of PVA-*g*-SBMA in different solvent.

Solvent	Ratio	Dissolved state	Illustration
Deionized water (DI water)	DI water: 10 ml<br>PVA-*g*-SBMA: 700 mg	Completely dissolved	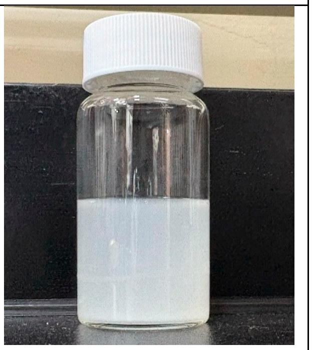
Dimethyl sulfoxide (DMSO)	DMSO: 10 ml<br>PVA-*g*-SBMA: 700 mg	Completely dissolved	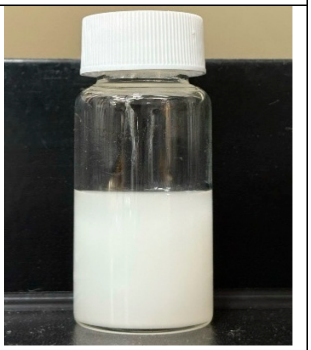

Table S2. Mn, Mw, and PDI results from GPC.

Sample	Elution Volume (ml)	Retention Time (min)	Adjusted RT (min)	Mn	Mw	MP	Mz	Mz+1	PDI
PVA-*g*-SBMA	6.737	6.737	6.737	15755	18439	19572	20791	22653	1.170
SBMA	7.943	7.943	7.943	2459	2724	2479	3089	3578	1.108

Table S3. Morphology and flexibility of PVA-*g*-SBMA films in different solvent.

Solvent	Ratio	Illustration
Deionized water<br>(DI water)	DI water: 10 ml<br>PVA-*g*-SBMA: 700 mg<br>PEDOT:PSS: 1 ml<br>GOPS: 0.1 ml	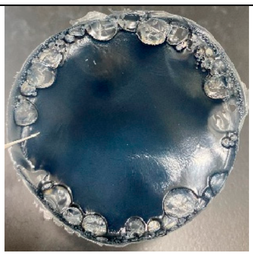
Dimethyl sulfoxide<br>(DMSO)	DMSO: 10 ml<br>PVA-*g*-SBMA: 700 mg<br>PEDOT:PSS: 1 ml<br>GOPS: 0.1 ml	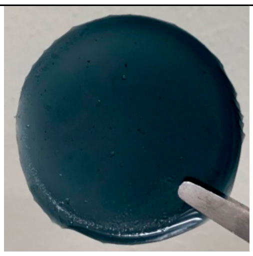<br>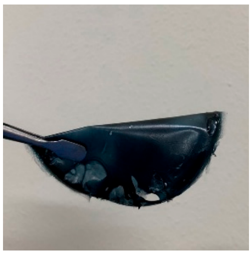

Table S4. Morphology of PVA-*g*-SBMA films at different crosslinking density.

GOPS amount	Morphology
0.01 wt%	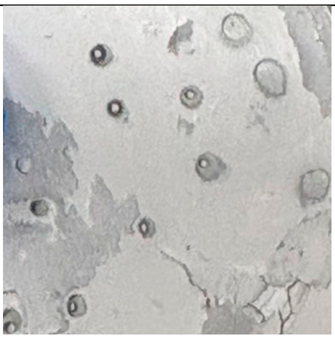
0.05 wt%	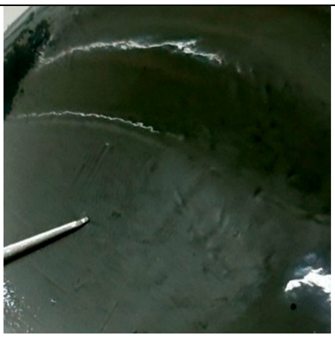
0.1 wt%	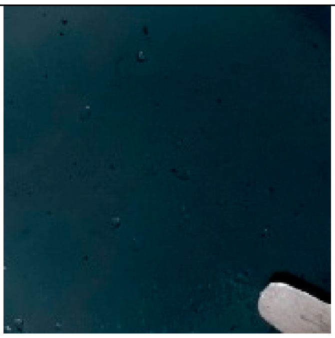

Table S5. Contact angle measurement results.

Sample	C0.05	C0.05 + LiTFSI 0.015 wt%	C0.05 + LiTFSI 0.02 wt%	C0.1	C0.1 + LiTFSI 0.015 wt%	C0.1 + LiTFSI 0.02 wt%
θ (°)	103.72 ± 0.15	101.33 ± 0.68	89.61 ± 1.88	67.59 ± 1.58	61.26 ± 1.28	43.21 ± 1.35

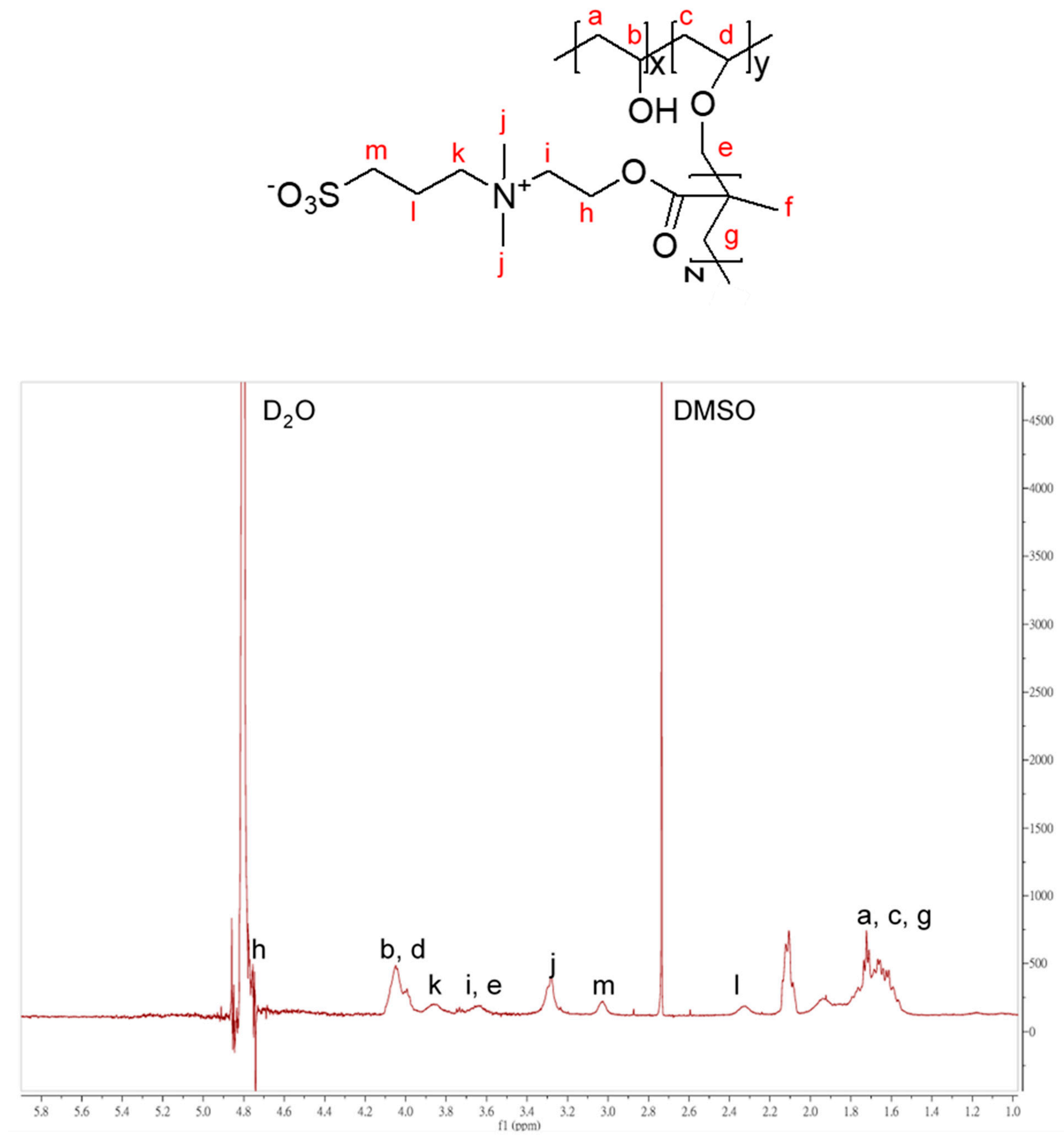

Figure S1. Protons assignment of PVA-*g*-SBMA and ¹H NMR spectrum of PVA-*g*-SBMA.

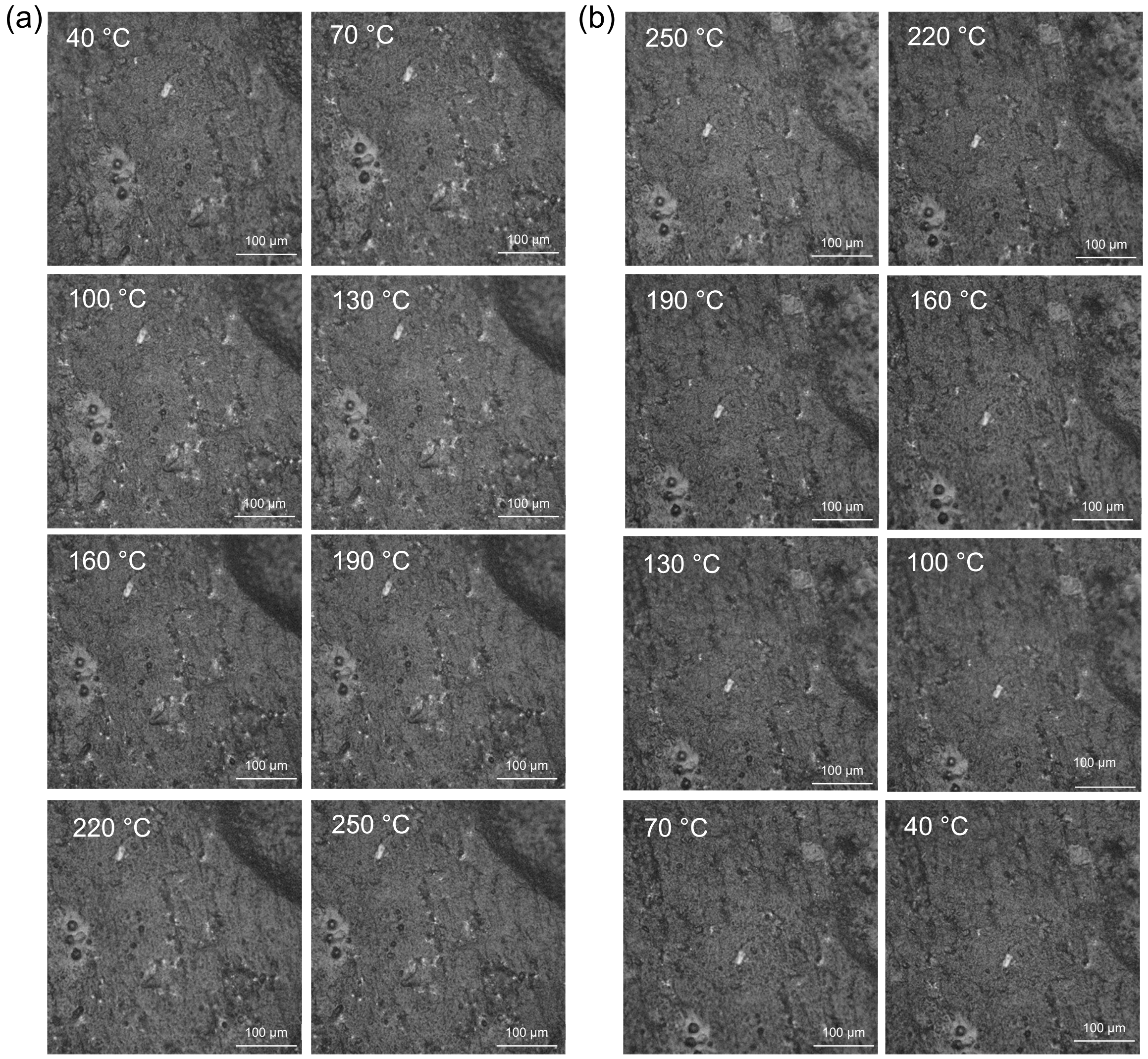

Figure S2. Polarized Optical Microscopy images of the C0.1 film under a heating and cooling rate of 10 °C/min: (a) during heating and (b) during cooling.

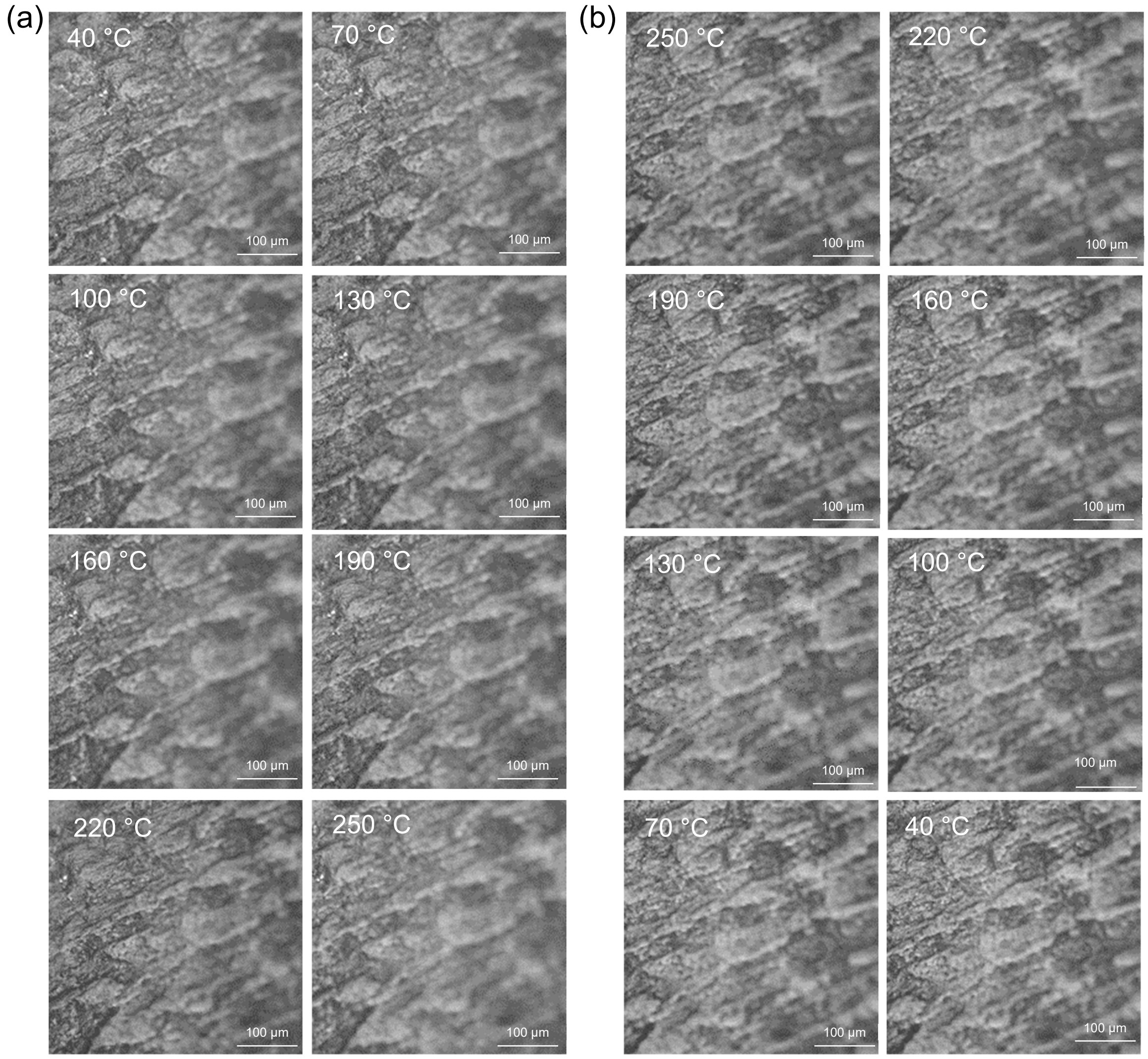

Figure S3. Polarized Optical Microscopy images of the C0.1 + LiTFSI 0.015 wt% film under a heating and cooling rate of 10 °C/min: (a) during heating and (b) during cooling.

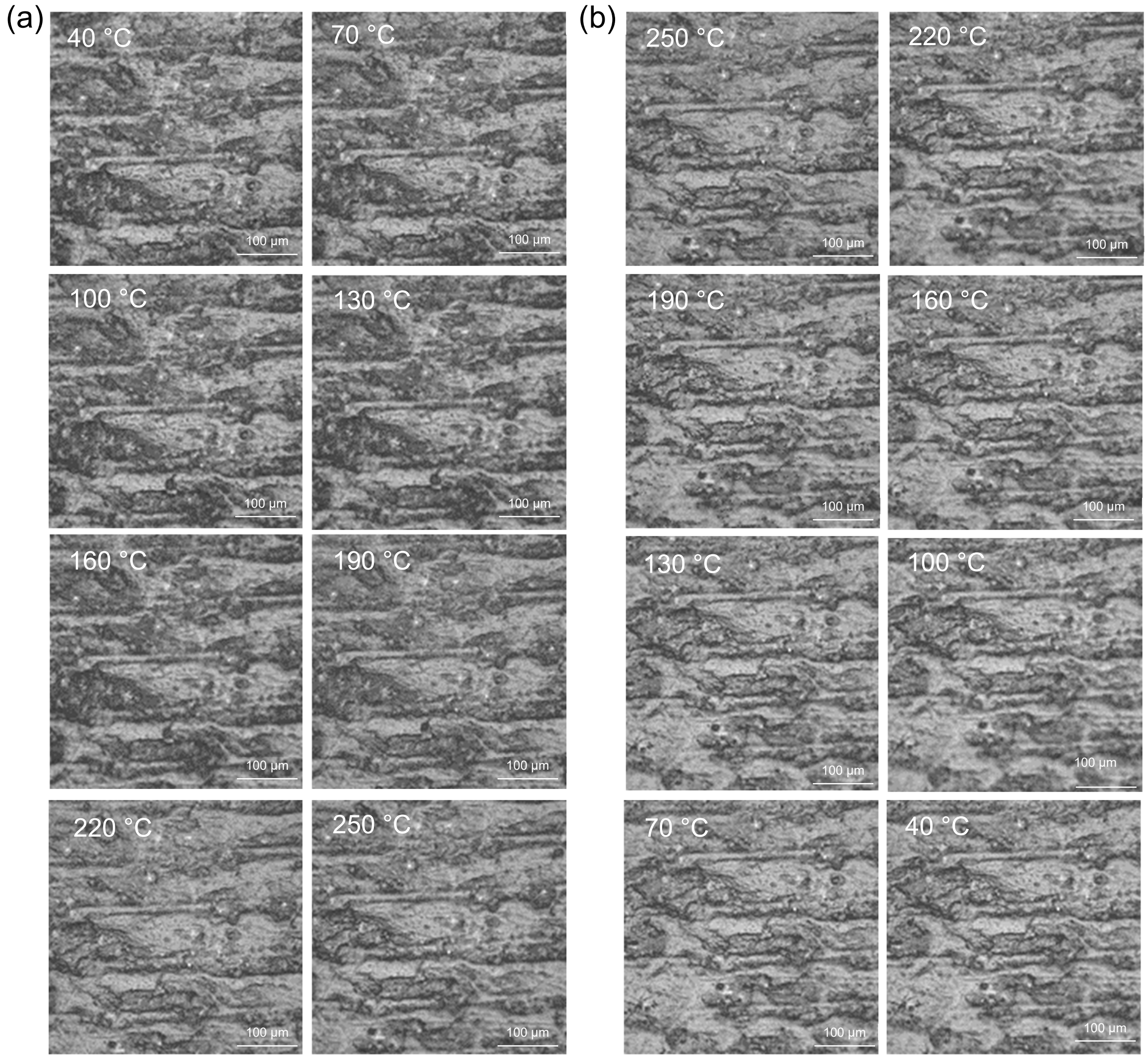

Figure S4. Polarized Optical Microscopy images of the C0.1 + LiTFSI 0.02 wt% film under a heating and cooling rate of 10 °C/min: (a) during heating and (b) during cooling.

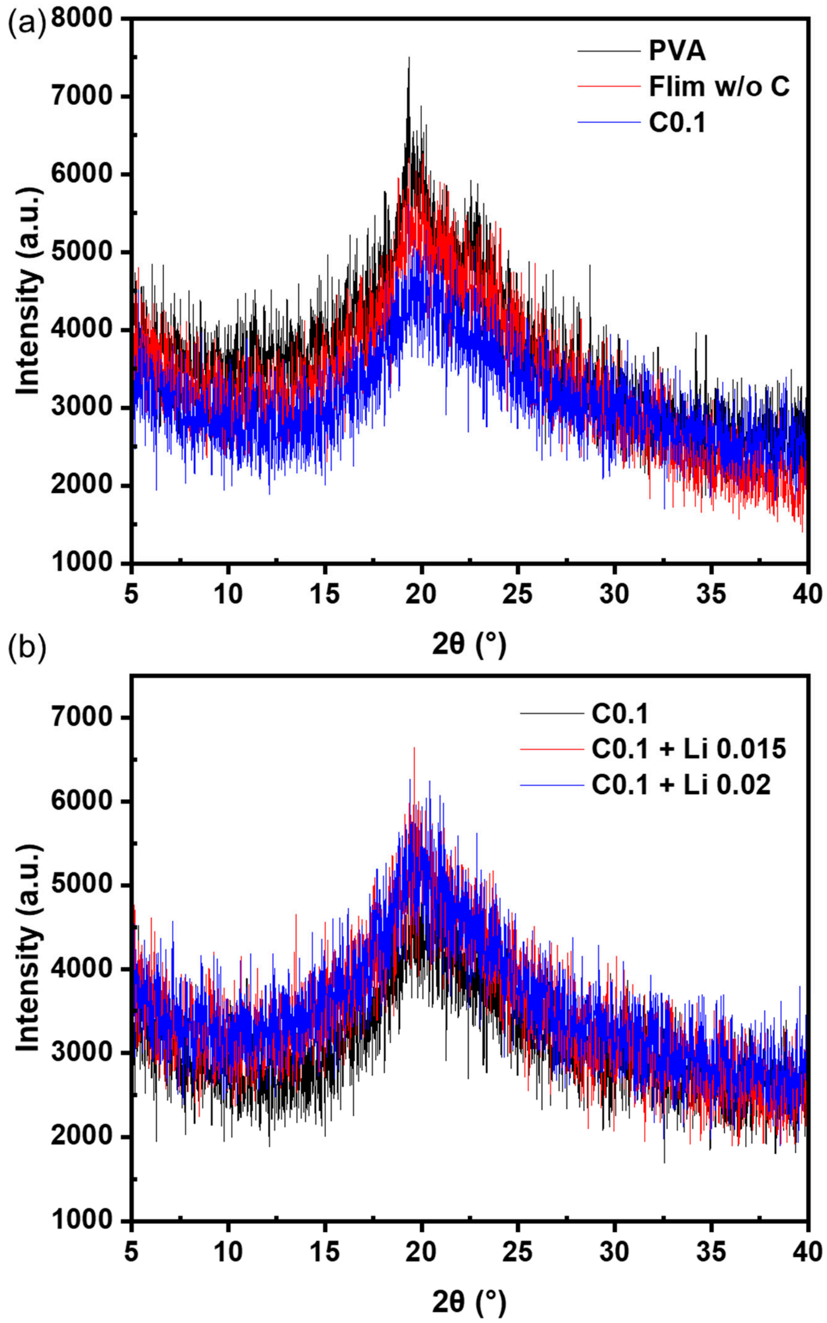

Figure S5. XRD patterns of (a) PVA, PVA-*g*-SBMA, and C0.1, and (b) the C0.1 system films.

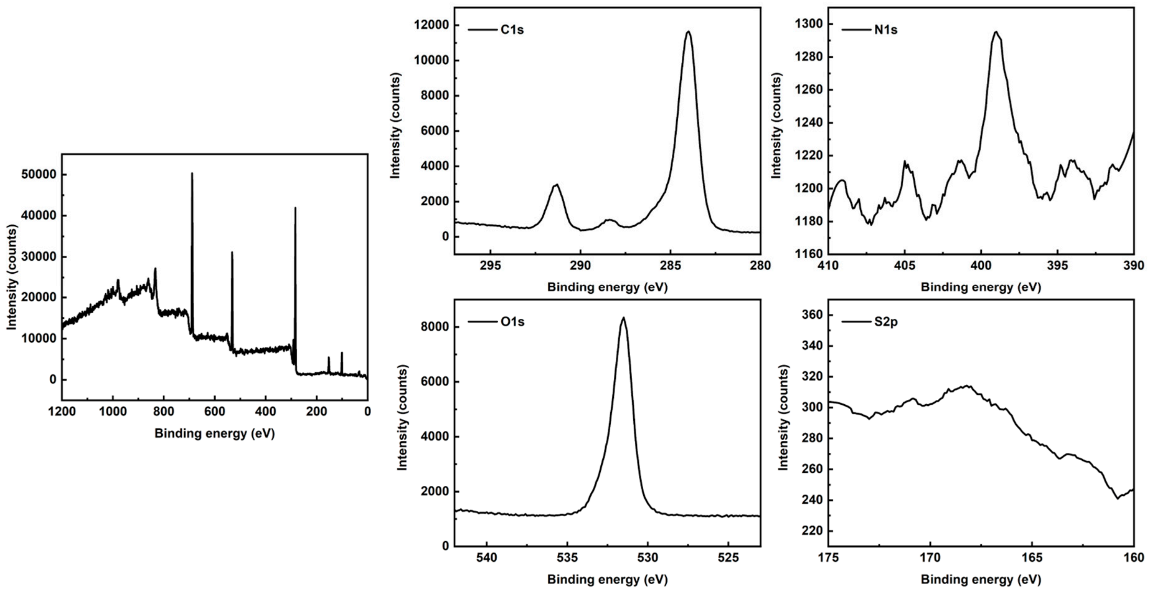

Figure S6. XPS spectra of PVA-*g*-SBMA and C0.1 film samples, with high-resolution core-level scans of C 1s, N 1s, O 1s, and S 2p.
